# Entecavir: A Review and Considerations for Its Application in Oncology

**DOI:** 10.3390/ph16111603

**Published:** 2023-11-14

**Authors:** Tânia Lourenço, Nuno Vale

**Affiliations:** 1 PerMed Research Group, Center for Health Technology and Services Research (CINTESIS), Rua Doutor Plácido da Costa, 4200-450 Porto, Portugal; taniadiaslourenco@hotmail.com; 2 CINTESIS@RISE, Faculty of Medicine, University of Porto, Alameda Professor Hernâni Monteiro, 4200-319 Porto, Portugal; 3 Faculty of Pharmacy, University of Porto, Rua de Jorge Viterbo Ferreira 228, 4050-313 Porto, Portugal; 4 Department of Community Medicine, Information and Health Decision Sciences (MEDCIDS), Faculty of Medicine, University of Porto, Rua Doutor Plácido da Costa, 4200-450 Porto, Portugal

**Keywords:** entecavir, antiviral drug, hepatitis B, KDM5B inhibitor, drug repurposing

## Abstract

Entecavir (ETV) is a drug used as a first-line treatment for chronic hepatitis B (CHB) virus infection because it is a guanosine nucleoside analogue with activity against the hepatitis B virus polymerase. The ETV dosage can range from 0.5 mg to 1 mg once a day and the most common side effects include headache, insomnia, fatigue, dizziness, somnolence, vomiting, diarrhea, nausea, dyspepsia, and increased liver enzyme levels. In addition to its conventional use, ETV acts as an inhibitor of lysine-specific demethylase 5B (KDM5B), an enzyme that is overexpressed in breast, lung, skin, liver, and prostate tumors and is involved in the hormonal response, stem cell regeneration, genomic stability, cell proliferation, and differentiation. The KDM5B enzyme acts as a transcriptional repressor in tumor suppressor genes, silencing them, and its overexpression leads to drug resistance in certain tumor types. Furthermore, the literature suggests that KDM5B activates the PI3K/AKT signaling pathway, while reducing KDM5B expression decreases AKT signaling, resulting in decreased tumor cell proliferation. In silico studies have demonstrated that ETV can inhibit tumor cell proliferation and induce apoptosis by reducing KDM5B expression. ETV also appears to inhibit PARP-1, has a high genetic barrier, reducing the chance of resistance development, and can also prevent the reactivation of the hepatitis B virus in cancer patients, which have proven to be significant advantages regarding its use as a repurposed drug in oncology. Therefore, ETV holds promise beyond its original therapeutic indication.

## 1. Background

According to the World Health Organization (WHO), in 2019, there were around 296 million people with CHB worldwide. It is worth noting that 95% of CHB cases are related to vertical or horizontal transmission during childhood, while the remaining 5% belong to CHB acquired in adulthood. The WHO also reported that, in 2019, hepatitis B caused nearly 820,000 deaths, the vast majority of which were a result of CHB cases leading to cirrhosis or liver tumors [1]. Interestingly, the number of people with CHB in China accounts for approximately half of the global population with CHB [2].

Due to the high mortality rates and large number of people affected by the disease, it was crucial to develop treatments for this illness. The first drug approved for CHB treatment was interferon-alpha, in 1991, followed by oral therapies such as lamivudine (1998), adefovir (2002), ETV (2005), telbivudine (2006), and tenofovir (2008). On the other hand, pegylated interferon approval took place in 2005 [3]. Regarding ETV, after its approval by the Food and Drug Administration (FDA), regulatory agencies in Europe, Japan, and China also granted approval. Currently, this drug is available in over 70 countries and is listed on the WHO List of Essential Medicines [4,5].

**Figure 1:**
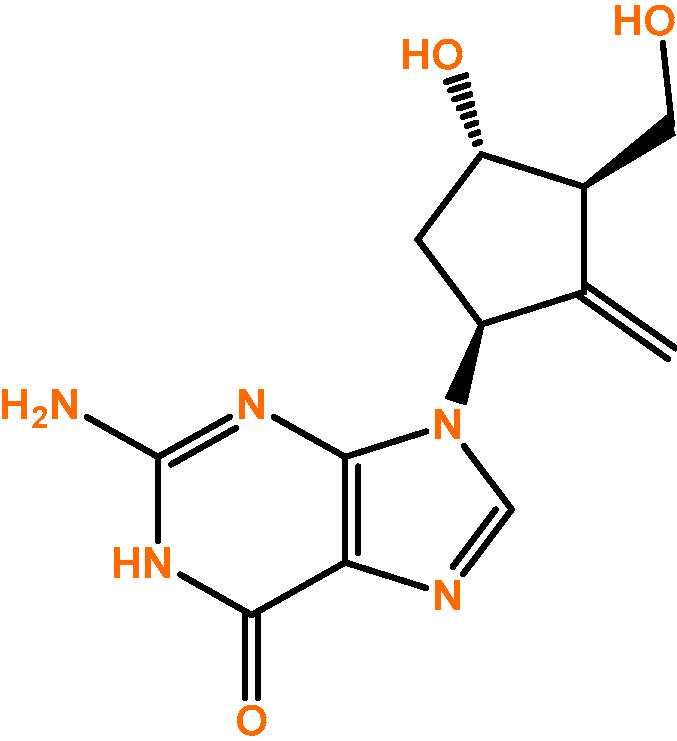
ETV chemical structure.

In 1995, Bristol-Myers Squibb found that the compound SQ-34676, previously developed to combat herpes simplex virus but proven ineffective, was useful in hepatitis B virus treatment, particularly in CHB. In fact, it was found to be the most potent compound discovered thus far. After this, this compound was named BMS 200475 and it is currently known as ETV (Figure 1) [5].

Thus, ETV has therapeutic indications for the treatment of hepatitis B virus infection, namely as a first-line treatment for CHB in adult and pediatric patients aged over 2 years. The treatment for pediatric patients should be based on a careful assessment of the individual patient’s needs, pediatric clinical guidelines, and baseline histological information. ETV is marketed under the brand name Baraclude^®^ by Bristol-Myers Squibb, and the recommended dosage is 0.5 mg or 1 mg once a day, available as a tablet and oral solution. The most common side effects include headache, insomnia, fatigue, dizziness, somnolence, vomiting, diarrhea, nausea, dyspepsia, and increased liver enzyme levels [6,7].

## 2. Search Strategy

The bibliographic research was initiated during May 2023, without any language restrictions, employing the PubMed and Google Scholar databases. A combination of keywords and appropriate terms for each database was employed, including “entecavir”, “chronic hepatitis B”, “antiviral drug”, “KDM5B”, “oncology”, “PARP”, “cancer”, “drug repurposing”, and “high genetic barrier”, among others. Publications dated up to 2023 were included, always selecting the most recent and current articles whenever possible. In addition to the selection based on the publication date, articles were also chosen based on their quality, rigor, and relevance. In addition to searching these databases, research was conducted on reputable websites, including the WHO, and the websites of national regulatory agencies for medicines (INFARMED—Portuguese National Authority for Medicines and Health Products) and international agencies (FDA—U.S. Food and Drug Administration).

Finally, the reference management software Mendeley Desktop version 1.19.8 was used to manage the references.

As previously mentioned, ETV has been commercially available as an antiviral drug since 2005 [3]; however, this review article highlights other pharmacological characteristics of ETV that may enable it to acquire new therapeutic indications, particularly in oncological treatment.

## 3. Pharmacological Properties

ETV is a guanosine nucleoside analogue with activity against the hepatitis B virus polymerase, so it is a drug with antiviral activity. Thus, ETV is intracellularly phosphorylated (Table 1), transforming into ETV-triphosphate (active form), which competes with deoxyguanosine triphosphate, a natural substrate of the hepatitis B virus reverse transcriptase. Consequently, ETV-triphosphate inhibits all stages of the viral replication process that rely on viral polymerase activity, namely the initiation of the hepatitis B virus polymerase, the reverse transcription of the negative strand of hepatitis B virus DNA from pregenomic mRNA, and the synthesis of hepatitis B virus positive strand DNA [6,7,8].

In general, ETV is well tolerated; however, due to its primarily renal excretion, it should be carefully coadministered with drugs that reduce renal function. Since it is not metabolized by cytochrome P450, interactions mediated by these enzymes are unlikely to occur [6].

## 4. Antiviral Resistance

The emergence of viral mutations or genotypic resistance is a situation that may occur after the prolonged administration of certain antiviral therapies, including treatments for CHB, especially with lamivudine and adefovir, which have a low genetic barrier to resistance. This situation is associated with increased levels of hepatitis B virus DNA and intracellular hepatic aminotransferase serum concentrations, leading to the exacerbation of liver disease, which could trigger acute liver failure and result in death [9,10].

Thus, mutations that lead to resistance development change the viral polymerase’s interaction with the drug, modifying the drug’s inhibitory effect on the hepatitis B virus polymerase [9]. Therefore, viral resistance is dependent on the virus’s ability to replicate in each environment, with mutant viruses having a survival advantage in the presence of antiviral drugs and exhibiting replication similar to that of non-mutant viruses. It also depends on the effectiveness of the antiviral drug and its ability to rapidly inhibit the hepatitis B virus polymerase. The genetic barrier to resistance is linked to the number of mutations that the virus needs to accumulate to replicate itself in the presence of the antiviral drug, so drugs with a high genetic barrier are less likely to develop resistance. Beyond these factors, the susceptibility of immunocompromised patients, non-adherence to therapy, challenges in achieving effective drug concentrations in obese people, individual enzymatic activity, and the prior use of antiviral drugs are also significant contributors to the resistance to antiviral therapy [10].

In this way, a few patients develop resistance to therapy after prolonged ETV treatment. In treatment-naïve patients, the ETV resistance rate after one year is 0%, and it remains below 1% after two years, proving that ETV exhibits a low antiviral resistance rate. This is likely related to three factors, namely its strong antiviral effect, the high genetic barrier to resistance, which means that multiple mutations are required for resistance development, and the impaired replication of resistant variants [5,10]. Indeed, resistance is more common in non-naïve patients, and the ETV resistance increases to 51% after 5 years in patients resistant to lamivudine treatment, but only 1.2% in treatment-naïve patients. For patients who become resistant, a combination therapy should be administered, such as ETV and tenofovir; in cases of ETV resistance, monotherapy with tenofovir is recommended [9]. It is worth noting that ETV resistance is higher in people resistant to lamivudine due to the primary presence of lamivudine resistance mutations and compensatory ETV resistance mutation development. However, it is not yet known whether ETV resistance can increase the rate of tenofovir-resistant mutations. Despite the high genetic barrier to resistance, ETV resistance increased from 6.04% to 15.02% between 2011 and 2017, likely due to the frequent use of antivirals with a low resistance barrier [2].

Therefore, in patients with a high initial viral load, it takes more time to achieve the desired treatment outcomes and to reduce the hepatitis B virus viral load, as the rate of viral suppression increases over time. Furthermore, it is known that people with a high initial hepatitis B virus viral load are more likely to develop resistance to ETV. Thus, it is important to emphasize the need to monitor the viral load in patients infected with the hepatitis B virus to better control the development of resistance [11].

## 5. PARP Inhibitor

According to Sherin et al., in silico studies have demonstrated that ETV could potentially be a candidate for breast, ovarian, and prostate cancer treatment, because it could inhibit the enzyme PARP1 that is expressed in these tissues, which plays a crucial role in DNA signaling and DNA damage repair [12]. Therefore, by inhibiting PARP in cancer cells, there would be the accumulation of unrepaired errors that ultimately lead to cell death [12,13].

In essence, the occurrence of DNA mutations can trigger tumorigenesis. Healthy cells have developed defense mechanisms within several molecular pathways known as the DNA damage response (DDR), which can identify and repair damage, contributing to genome integrity. The DDR encompasses the repair of double-strand DNA damage breaks (DSB), including homologous recombination (HR) and non-homologous recombination, as well as the repair of single-strand breaks (SSB), which includes base excision repair (BER). Proteins such as BRCA1, BRCA2, PALB2, ATM, CHEK1, CHEK2, and RAD51 are involved in the HR repair process, while poly(ADP-ribose) polymerases 1 and 2, referred to as PARP1 and PARP2, are associated with the BER, with some of these proteins being frequently mutated in cancer patients. PARP not only act as regulators of the identification and repair process for SSB through BER, but also play a role in repairing DSB because they promote the activation of the repair by HR while inhibiting less conservative repair pathways such as the non-homologous route end joining (NHEJ) [13,14].

Furthermore, PARP inhibitors operate through the synthetic lethality principle, where the loss of function in two genes results in cell death, while the loss of function in just one of them is compatible with cell viability. In other words, cells with dysfunctional BRCA1 and BRCA2 genes are unable to repair DNA damage and depend on PARP to detect and activate alternative repair pathways, becoming particularly vulnerable to PARP inhibitors. In this way, PARP inhibitors are indicated for the treatment of tumors with BRCA1/2 gene mutations or tumors with BRCAness, as they exhibit deficits in HR repair. Although the functional loss of one of the genes BRCA or PARP is compatible with cell viability, inhibiting PARP in cells with BRCA mutations renders them incapable of repairing DNA damage, leading to the accumulation of errors that ultimately lead to cell death [13,14]. Therefore, if it is indeed confirmed that ETV acts as a PARP inhibitor, it will always be relevant to conduct a genetic study of the patients to analyze the BRCA1 and BRCA2 genes.

Interestingly, the literature indicates that, in addition to ETV, the antiviral drugs didanosine, acyclovir, valganciclovir, penciclovir, ganciclovir, and valacyclovir have also shown potential in in silico studies as candidates for the treatment of breast, ovarian, and prostate tumors, as they inhibit PARP1 [12]. For this reason, we postulate that these drugs may also act through the principle of synthetic lethality, since they will only be useful in the treatment of tumors with BRCA1/2 mutations or in BRCAness tumors that, in combination with PARP inhibition, are unable to repair DNA damage, leading to cell death. However, further confirmation is needed. On the other hand, the use of radiation (radiotherapy) is one of the therapeutic approaches employed in several tumors. Radiation blocks cell division and proliferation, ultimately leading to apoptosis through SSB and DSB. In addition, it has been recognized that there are radiosensitizing agents that, when combined with radiation, result in greater tumor inactivation and are, therefore, beneficial for oncological treatment [15].

Indeed, the existence of antiviral agents with radiosensitizing properties is already documented in the literature, such as cidofovir, which selectively radiosensitizes cells infected with human papillomavirus and saquinavir [16,17]. In addition to the relevant characteristics mentioned regarding ETV, it would be useful to determine whether ETV is a radiosensitizing molecule and to assess whether its ability to target DDR in tumor cells increases its radiosensitivity compared to normal cells. If ETV does not exhibit radiosensitizing properties, it can also be combined with another radiosensitizing agent.

## 6. ETV before Initiating Chemotherapy

Hepatitis B virus reactivation after chemotherapy or immunosuppressive treatment can occur in people who are already cured or have CHB. Reactivation can happen in cases of both hematological and solid tumor treatment, and it is related to various factors, including patient characteristics (immunity, gender, age, pre-existing diseases), viral characteristics, and the drugs used (high risk: anti-CD20 and anti-CD52 monoclonal antibodies, systemic chemotherapy; moderate risk: tyrosine kinase inhibitors, corticosteroids administered for more than 4 weeks; low risk: other immunosuppressants and corticosteroids administered for less than 4 weeks). People with breast cancer treated with anthracyclines, as well as those with rheumatological and inflammatory diseases treated with immunosuppressants or biological drugs, and patients treated with B-cell-depleting agents like rituximab and ofatumumab, are at reactivation risk [18,19,20]. According to Zhang et al., treatments with programmed cell death protein 1/programmed death ligand 1 (PD-1/PDL-1) inhibitors also deserve special attention, as they may lead to hepatitis B virus reactivation [20].

In this way, hepatitis B virus reactivation can occur at the beginning of chemotherapy/immunosuppressive therapy (within the first two weeks) or up to a year after its cessation. This situation is a cause for concern, as it may result in treatment complications, disease progression, and potentially fatal acute liver failure. Therefore, the development of strategies to prevent hepatitis B virus reactivation in these patients is crucial. Subsequently, it is essential to assess patients before initiating chemotherapy or immunosuppressive treatments. Given the impossibility of screening all individuals, it is necessary to attempt to screen those at high or moderate risk of reactivation, and then initiate antiviral prophylactic treatment to prevent the reactivation of the hepatitis B virus. ETV can be a viable option due to its high genetic barrier. It has demonstrated greater efficacy than lamivudine in terms of hepatitis risk (0% vs. 13.3%), hepatitis B virus reactivation (6.6% vs. 30.0%), and chemotherapy discontinuation (1.6% vs. 18.3%). In essence, ETV prophylaxis not only reduces the risk of reactivation but also minimizes the occurrence of viral hepatitis and the likelihood of chemotherapy interruption; however, it is still necessary to evaluate the optimal duration of prophylactic antiviral therapy [18,19].

## 7. KDM5B Inhibitor as a Therapeutic Target

**Scheme 1:**
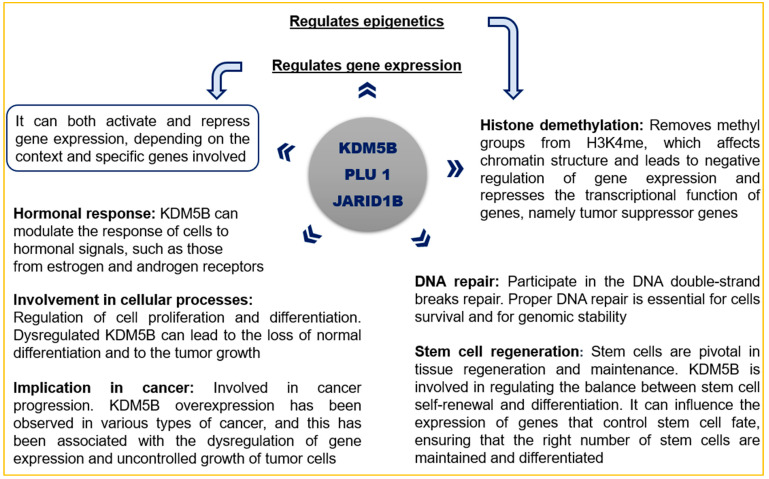
Importance of KDM5B in cancer.

Specific lysine histone demethylases (KDM5), such as KDM5B, play a crucial role in gene expression and epigenetic regulation in several types of cancer (Scheme 1). They are essential in transcriptional processes by controlling the methylation of histone H3 lysine 4 (H3K4). KDM5B, also known as PLU1 or JARID1B, is an enzyme that is overexpressed in several tumors, and it is involved in the hormonal response, DNA repair, stem cell regeneration, genomic stability, cell proliferation, and differentiation. KDM5B demethylates H3K4 and acts as a transcriptional repressor on tumor suppressor genes, leading to their inactivation [21,22]. Furthermore, KDM5B overexpression contributes to drug resistance in certain tumor types and induces metastasis and angiogenesis by repressing HOXA5 [23,24].

### 7.1. KDM5B Importance in Tumoral Process

#### 7.1.1. Breast Cancer

KDM5B is related to tumors with worse prognoses, therapy resistance, inflammatory responses, metastasis development, and the proliferation of breast cancer cells through the repression of tumor suppressors, namely BRCA1, HOXA5, and CAV1. Moreover, the KDM5B-NTT isoform is present in breast cancer cells and the TFAP2C-Myc-KDM5B complex is associated with the repression of p21, tumorigenesis, and therapeutic failure. Besides this, inhibiting KDM5B induces HEXIM1 (tumor suppressor) expression, leading to the inhibition of tumor cell proliferation and the prevention of metastasis formation. KDM5B represses the tumor suppressor miRNA let-7e and activates cyclin D1, and KDM5B interacts with EMSY and suppresses miR-31 by demethylating H3K4me3. It also interacts with estrogen receptors and promotes the tumor growth of ER+ cancers. Generally, luminal breast cancers express higher levels of KDM5B, while triple-negative breast cancers express lower levels of KDM5B when compared to ER+ cancers. In addition, HER2+ tumors and luminal tumors are sensitive to KDM5 inhibition, and KDM5 inhibitors exhibit a synergistic effect with trastuzumab and lapatinib. Interestingly, in triple-negative breast cancers, there are differing opinions regarding the role of KDM5B. Some researchers suggest that it may act as a tumor suppressor, while others claim that it promotes metastasis and the progression of this tumor type [21,22,24,25,26].

#### 7.1.2. Lung Cancer

Lung tumor tissues exhibit high expression of KDM5B, and the decreased expression of KDM5B leads to the slowing down of tumor growth through the E2F/RB1 pathway. KDM5B is associated with resistance to the drugs gefitinib, cisplatin, and doxorubicin. Besides this, KDM5B is associated with cell proliferation and invasion, the negative regulation of p53, the epithelial–mesenchymal transition process (increased ZEB1 and ZEB2 and decreased miR-200), and, consequently, tumor cell migration and metastasis development (particularly brain metastases in non-small-cell lung cancer). Moreover, in non-small-cell lung cancer, KDM5B activates the c-MET pathway, leading to worse prognoses and triggering resistance to radiation treatment. However, there are conflicting studies: H3K4me3 decreases DNA damage repair; KDM5B facilitates DNA repair in tumor cells under radiation treatment; and KDM5B inhibition impairs DNA damage repair, sensitizing tumor cells to radiation [21,22,27,28].

#### 7.1.3. Skin Cancer

The influence of KDM5B in melanoma is not yet clear, as some studies suggest that KDM5B is associated with tumor cells and metastases, while others indicate that KDM5B acts as a tumor suppressor by regulating retinoblastoma proteins. However, KDM5B is overexpressed in benign tumors and in slowly progressing tumors in patients who do not respond to immune checkpoint inhibitors. On the other hand, KDM5B is minimally expressed in aggressive and metastatic tumors. KDM5B is associated with therapeutic resistance, and reducing its expression increases the sensitivity of melanocytes to treatment. Moreover, the presence of KDM5B isoforms can contribute to tumor progression, while the RBP2-H1 isoform is linked to poor prognoses and KDM5B reduces intratumoral heterogeneity and is related to oxidative phosphorylation [21,22,25,28].

#### 7.1.4. Stomach Cancer

KDM5B is overexpressed in gastric tumors, being related to tumor proliferation, migration, and invasion, with reduced immune cell infiltration within the tumor, and it is associated with poor prognostic outcomes, functioning as a tumor promoter. miR-194 and miR-29c reduce KDM5B expression and decrease proliferation and tumor growth, while Helicobacter pylori reduces miR-29c, leading to KDM5B overexpression. KDM5B is regulated by the transcription factors E2F1, p53, Sp1, and Sp3. On the other hand, KDM5B overexpression is associated with cisplatin resistance. Moreover, KDM5B is associated with metastasis development through the Akt pathway, and Akt inhibitors reduce gastric tumor cell migration. KDM5B increases CCND1 (a cell-cycle-regulatory protein that promotes cell proliferation and is associated with tumorigenesis) expression through H3K27me3, and decreasing KDM5B prevents CCND1 expression. Inhibiting NEK2 results in a KDM5B decrease and consequently a H3K4me3 increase. Conversely, NEK2 overexpression leads to an KDM5B increase and H3K4me3 decrease, suggesting that NEK2 may regulate KDM5B expression and H3K4me3 through the β-catenin/Myc pathway. The signaling pathway NEK2/β-catenin/Myc/KDM5B/H3K4me3 plays a significant role in carcinogenesis [21,29,30,31].

#### 7.1.5. Bladder Cancer

KDM5B regulates the cell cycle of bladder tumor cells and an inverse relationship between KDM5B and connexin 26 is observed; KDM5B is overexpressed, while connexin 26 is underexpressed. KDM5B inhibition increases immunogenicity in bladder tumor cells with FGFR3 mutation [21,25,32].

#### 7.1.6. Liver Cancer

KDM5B is overexpressed in hepatocellular carcinomas, especially those associated with hepatitis B virus. It is related to the epithelial–mesenchymal transition process, metastasis development, tumor proliferation, a worse prognosis, and decreased overall survival. Moreover, KDM5B expression is related to the tumor size, staging, and degree of differentiation. Additionally, higher expression of KDM5B is associated with the reduced inhibition of miR-448. KDM5B contributes to hepatocellular carcinoma progression through the miR-448/YTHDF3/ITGA6 axis, and KDM5B inactivates PTEN, while silencing KDM5B reduces tumor cell proliferation and induces cell cycle arrest. Besides this, KDM5B overexpression triggers AKT signaling and KDM5B downregulation reduces AKT signaling. The depletion of KDM5B inhibits cell proliferation through the regulation of the p15 and p27 proteins and an increase in the trimethylation of H3K4. In vivo clinical trials revealed that KDM5B and KDM5C were involved in the development of alcohol-induced fibrosis in female mice through the negative regulation of the aryl hydrocarbon receptor in hepatic stellate cells, while males developed fibrosis through a different mechanism. In this case, the decrease in KDM5B and KDM5C led to increased aryl hydrocarbon receptor expression, Arnt, and Aip in female mice, preventing fibrosis. ETV (KDM5B inhibitor) significantly improves the hepatic histopathology and reduces CHB progression, decreasing the likelihood of hepatocellular carcinoma development and increasing the survival rate of patients with hepatitis-virus-related liver tumors [5,21,23,28,33,34,35].

#### 7.1.7. Colorectal Cancer

KDM5B is overexpressed in this type of tumor and is associated with tumor progression and poor prognosis. Reducing KDM5B leads to apoptosis and inhibits the migration, invasion, and proliferation of HT-29 tumor cells and increases the trimethylation of H3K4 in the p16/INK4A region, which suppresses the tumor. In vivo, KDM5B downregulation also reduced tumor growth. KDM5B overexpression decreases CCL14 and activates the Wnt/β-catenin pathway, promoting tumor progression. Conversely, KDM5B inhibits CDX2 expression, which is crucial for the signaling of the Wnt/β-catenin pathway, through H3K4me3 demethylation. NEK2 overexpression is associated with the lower expression of β-catenin at the cell membrane and the accumulation of β-catenin in the cytoplasm and nuclei of tumor cells [21,31,36,37].

#### 7.1.8. Prostate Cancer

The use of microRNAs in regulating KDM5B was evaluated, and it was found that the expression of miR-29a inhibited the proliferation of prostate cancer cells and induced their apoptosis by decreasing the expression of KDM5B. Additionally, miR-137 functions as a tumor suppressor in prostate cancer. H3K4me3 is associated with prostatic carcinogenesis, and Skp2 inactivation resulted in decreased H3K4me3 levels, subsequently reducing cell proliferation and migration. KDM5B has been detected in metastatic adenocarcinomas and neuroendocrine tumors and is associated with advanced tumors, a poor prognosis, and the viability of tumor cells. The tumor suppressor gene PTEN is frequently mutated in various types of cancer, as it functions as a phosphatase that dephosphorylates phosphatidylinositol 3,4,5-trisphosphate (PIP3) into phosphatidylinositol 4,5-bisphosphate (PIP2). The rate of PIP3/PIP2 is regulated not only by PTEN activity but also by PI3K. The loss of PTEN function leads to PIP3 accumulation in the cell membrane and increased activity of the PI3K/AKT pathway, which disrupts multiple signaling pathways. The literature suggests that KDM5B activates the PI3K/AKT signaling pathway, while reducing KDM5B expression decreases AKT signaling and significantly lowers P110α (a subunit of PI3K) and PIP3 levels, ultimately resulting in the reduced proliferation of prostate cancer cells and suppression of tumorigenesis. Moreover, KDM5B is overexpressed in prostate tumor tissues, especially in mice that do not express PTEN and exhibit hyperactivation of the AKT pathway due to the absence of PTEN. KDM5B binds to androgen receptors (essential for prostate cancer development/progression) and enhances their transcriptional activity. Furthermore, 5–6% of individuals with prostate cancer exhibit amplification of the KDM5B copy number, and less than 1% of them show the heterozygous deletion of KDM5B [21,22,23,24,28,38].

#### 7.1.9. Others

KDM5B is also overexpressed in acute lymphoblastic leukemia, head and neck cancer, esophageal cancer, kidney cancer, ovarian cancer, neuroblastoma, Ewing sarcoma, glioma, and chronic myeloid leukemia, among others. In most of these cases, KDM5B also plays a significant role in metastasis development and tumor cell proliferation, while its silencing reduces cell growth [21,22,28].

Beyond ETV, the literature suggests that there are other antiviral drugs that could inhibit KDM5B, such as decitabine, abacavir, penciclovir, and 3-deazaneplanocin A, which show promising results in molecular docking studies [18]. Similarly to ETV, penciclovir is mentioned as an inhibitor of PARP1 and an inhibitor of KDM5B [12,21]. Therefore, with these two characteristics, it may be one of the strongest candidates for repurposing in oncological disease treatment, alongside ETV.

### 7.2. Repurposed ETV

KDM5B can be a useful target in many tumors’ treatment. Interestingly, ETV, beyond its antiviral activity, acts as an inhibitor of KDM5B; therefore, it has the potential to become a valuable drug in the fight against cancer, so ETV can be repurposed, as demonstrated in silico studies [21]. Since drug repurposing is an approach that involves identifying and developing new therapeutic indications for existing drugs, particularly in the field of oncology, this approach focuses on selecting non-anticancer drugs that, when used as a monotherapy or in combination with an anticancer agent, display therapeutic effects in treating cancer [39,40,41,42,43,44,45,46].

Drug repurposing offers several advantages, such as the existing knowledge about the drug, including its mechanisms of action, molecular targets, pharmacokinetic and pharmacodynamic properties, dosage, adverse effects, toxicological and pharmacovigilance data, safety information, production, and manufacturing methods, as well as insights from clinical trials and previous clinical use for original therapeutic indications. This allows the utilization of an existing drug, bypassing the need to search for new molecules and develop entirely new molecules, which could take several years until being commercialized [39,41,46]. As a result, the costs associated with researching these drugs are reduced, as the time required for the regulatory approval of the new therapeutic indication is significantly shortened [40,41,46]. Another advantage is the widespread availability of these drugs, with many of them included in the WHO List of Essential Medicines [41]. Moreover, the use of non-anticancer repurposed drugs for cancer treatment may decrease the adverse effects often associated with most anticancer medications, thereby enhancing the cancer patients’ quality of life [39], and, as the literature suggests, using repurposed drugs in combination therapies triggers a synergistic effect, enabling the reduction of individual drug concentrations while optimizing the therapy and increasing its success rate. Consequently, due to the different mechanisms of action within combination therapies, it becomes possible to minimize/delay the development of treatment resistance [41,46,47].

A combination therapy can use ETV with other cancer treatments, such as chemotherapy or targeted therapies. In an oral solution, the liquid form of ETV can be measured and administered in combination with other drugs used in the first-line treatment of cancer. The choice of formulation may depend on the patient’s specific needs, preferences, and any underlying medical conditions they may have.

Finally, the literature mentions that the IC_50_ values of ETV range from 0.36 nM to 3.6 nM concerning the inhibition of the hepatitis B virus in vitro. However, it is imperative to determine the IC_50_ values in tumor cell lines and, subsequently, in vivo, as well as shedding light on the mechanistic details of ETV’s action against various cancers [48]. Therefore, we believe that the IC_50_ of ETV for tumor cells will be significantly higher, requiring higher concentrations of ETV to inhibit tumor cells than to inhibit the hepatitis B virus.

## 8. Conclusions

In addition to its antiviral effect, ETV has emerged as a promising drug for oncology treatment. ETV has the potential to be beneficial in treating various types of cancer, particularly due to its ability to inhibit KDM5B and potentially PARP-1 as well. Furthermore, its high genetic barrier adds to its appeal for repurposing as an oncological agent. These are some of the points that we highlight as ETV’s strengths.

Overall, KDM5B emerges as a potential therapeutic target for oncological treatment due to its involvement in tumorigenesis, tumor progression, and resistance to therapy. The inhibition of KDM5B by ETV could potentially slow down or block these processes, making it a promising avenue for intervention. Similarly, if ETV indeed inhibits PARP, which play a role in DNA damage response and repair, it could trigger apoptosis in tumor cells. Furthermore, ETV has demonstrated the potential to prevent hepatitis B virus reactivation in cancer patients receiving immune checkpoint inhibitors, chemotherapy, and/or corticosteroids.

As a weakness, we point out the possibility that ETV alone may not be sufficient for oncological treatment and may require combination with another drug. However, the opportunities outweigh this weakness, as drug repurposing avoids the development of new molecules, and the combination of two therapeutic agents may yield superior results, such as reducing the individual drug concentrations and consequently the side effects, optimizing therapy, and delaying the drug resistance. On the other hand, the major obstacle will be the time that it may take to implement ETV as a repurposed drug in clinical practice. Indeed, it was not possible to find quantitative evidence from in vitro/in vivo studies in which ETV was used as a repurposed drug in cancer treatment. There are only a few in silico studies that mention the potential benefits and mechanisms of action that make ETV a good candidate for repurposing in oncology. Due to all the points mentioned above, and other drug repurposing studies involving antiviral drugs in oncology, we believe that it has strong potential. For this reason, we have already initiated in vitro combination studies with ETV and chemotherapeutic agents on tumor cell lines.

There are numerous pieces of evidence that support ETV’s potential for repurposing in cancer treatment. Initiating in vitro tests to determine the efficacy and safety of ETV in these contexts is crucial. Afterward, introducing this repurposed drug into clinical practice for oncological treatment could become a significant step forward in cancer therapy. It is imperative to accelerate research concerning this promising drug, allowing it to be tested in clinical practice as soon as possible. While these theories hold promise, further studies are needed to confirm their validity.

## Figures and Tables

**Table 1:** ETV pharmacokinetic properties.

Pharmacokinetics	Description	Ref.
Absorption	Rapidly absorbed Peak plasma concentrations: 0.5–1.5 h Bioavailability: at least 70% Steady state: 6–10 days after once daily administration Tablet and oral solution are bioequivalent	[6,7]
Distribution	Extensively distributed in tissues Low binding to human serum proteins: 13%	
Biotransformation	Is not a substrate, inhibitor, or inducer of CYP450 Phosphorylated to the active triphosphate form	
Elimination	Predominantly by the kidney Involves glomerular filtration and tubular secretion Phosphorylated metabolite half-life: 15 h	

## Data Availability

Not applicable.

## References

[1] Hepatitis B. https://www.who.int/news-room/fact-sheets/detail/hepatitis-b.

[2] Shang J., Zhou J., Liu H., Ise R.M., Tu Y., Ran J., Bai L., Tang H. (2021). Efficacy of Different Nucleoside Analog Rescue Therapies for Entecavir-Resistant Chronic Hepatitis B Patients. BMC Infect. Dis..

[3] Prifti G.M., Moianos D., Zoidis G., Giannakopoulou E., Pardali V., Tavis J.E., Grigoris Z. (2021). Recent Advances in Hepatitis b Treatment. Pharmaceuticals.

[4] Lee K.S., Kweon Y.O., Um S.H., Kim B.H., Lim Y.S., Paik S.W., Heo J., Lee H.J., Kim D.J., Kim T.H. (2017). Efficacy and Safety of Entecavir versus Lamivudine over 5 Years of Treatment: A Randomized Controlled Trial in Korean Patients with Hepatitis B e Antigen-Negative Chronic Hepatitis B. Clin. Mol. Hepatol..

[5] Tang H., Griffin J., Innaimo S., Lehman-Mckeeman L., Llamoso C. (2013). The Discovery and Development of a Potent Antiviral Drug, Entecavir, for the Treatment of Chronic Hepatitis B. J. Clin. Transl. Hepatol..

[6] Baraclude. https://www.ema.europa.eu/en/documents/product-information/baraclude-epar-product-information_pt.pdf.

[7] Entecavir. https://pubchem.ncbi.nlm.nih.gov/compound/Entecavir.

[8] Kim D.Y., Kim J.H., Tak W.Y., Yeon J.E., Lee J.H., Yoon J.H., Lee Y.J., Lee B.S., Han B.H., Lee H.C. (2017). Baracle® vs. Baraclude® for 48 Weeks in Patients with Treatment-Naïve Chronic Hepatitis B: A Comparison of Efficacy and Safety. Drug Des. Devel. Ther..

[9] Lim Y.S. (2017). Management of Antiviral Resistance in Chronic Hepatitis B. Gut Liver.

[10] Ghany M.G., Doo E.C. (2009). Antiviral Resistance and Hepatitis B Therapy. Hepatology.

[11] Takayama H., Komura T., Kagaya T., Sugimoto S., Orita N., Asahina Y., Nishikawa M., Ohta H., Kaneko S., Unoura M. (2021). Clinical Features and Resistance to Entecavir Monotherapy of Patients with Hepatitis B. Can. J. Gastroenterol. Hepatol..

[12] Sherin D.R., Manojkumar T.K. (2021). Exploring the Selectivity of Guanine Scaffold in Anticancer Drug Development by Computational Repurposing Approach. Sci. Rep..

[13] Branco C., Paredes J. (2022). PARP Inhibitors: From the Mechanism of Action to Clinical Practice. Acta Med. Port..

[14] Congregado B., Rivero I., Osmán I., Sáez C., López R.M. (2022). PARP Inhibitors: A New Horizon for Patients with Prostate Cancer. Biomedicines.

[15] Gong L., Zhang Y., Liu C., Zhang M., Han S. (2021). Application of Radiosensitizers in Cancer Radiotherapy. Int. J. Nanomed..

[16] Deutsch E., Haie-Meder C., Bayar M.A., Mondini M., Laporte M., Mazeron R., Adam J., Varga A., Vassal G., Magné N. (2016). Phase I Trial Evaluating the Antiviral Agent Cidofovir in Combination with Chemoradiation in Cervical Cancer Patients. Oncotarget.

[17] Pereira M., Vale N. (2022). Repurposing Alone and in Combination of the Antiviral Saquinavir with 5-Fluorouracil in Prostate and Lung Cancer Cells. Int. J. Mol. Sci..

[18] Ekpanyapong S., Reddy K.R. (2020). Hepatitis B Virus Reactivation: What Is the Issue, and How Should It Be Managed?. Clin. Liver Dis..

[19] Shih C.A., Chen W.C. (2021). Prevention of Hepatitis B Reactivation in Patients Requiring Chemotherapy and Immunosuppressive Therapy. World J. Clin. Cases.

[20] Zhang X., Zhou Y., Chen C., Fang W., Cai X., Zhang X., Zhao M., Zhang B., Jiang W., Lin Z. (2019). Hepatitis B Virus Reactivation in Cancer Patients with Positive Hepatitis B Surface Antigen Undergoing PD-1 Inhibition. J. Immunother. Cancer.

[21] Jose A., Shenoy G.G., Rodrigues G.S., Kumar N.A.N., Munisamy M., Thomas L., Kolesar J., Rai G., Rao P.P.N., Rao M. (2020). Histone Demethylase KDM5B as a Therapeutic Target for Cancer Therapy. Cancers.

[22] Ohguchi Y., Ohguchi H. (2022). Diverse Functions of KDM5 in Cancer: Transcriptional Repressor or Activator?. Cancers.

[23] Li G., Kanagasabai T., Lu W., Zou M.R., Zhang S., Celada S.I., Izban M.G., Liu Q., Lu T., Ballard B.R. (2020). KDM5B Is Essential for the Hyper-Activation of PI3K/AKT Signaling in Prostate Tumorigenesis. Cancer Res..

[24] Yoo J., Kim G.W., Jeon Y.H., Kim J.Y., Lee S.W., Kwon S.H. (2022). Drawing a Line between Histone Demethylase KDM5A and KDM5B: Their Roles in Development and Tumorigenesis. Exp. Mol. Med..

[25] Di Nisio E., Licursi V., Mannironi C., Buglioni V., Paiardini A., Robusti G., Noberini R., Bonaldi T., Negri R. (2023). A Truncated and Catalytically Inactive Isoform of KDM5B Histone Demethylase Accumulates in Breast Cancer Cells and Regulates H3K4 Tri-Methylation and Gene Expression. Cancer Gene Ther..

[26] Montano M.M., Yeh I.J., Chen Y., Hernandez C., Kiselar J.G., De La Fuente M., Lawes A.M., Nieman M.T., Kiser P.D., Jacobberger J. (2019). Inhibition of the Histone Demethylase, KDM5B, Directly Induces Re-Expression of Tumor Suppressor Protein HEXIM1 in Cancer Cells. Breast Cancer Res..

[27] Kaur N., Saini A., Walia A., Saxena R., Mathur P., Jha V. (2023). The Role of Tumor Suppressor Gene Epigenetic Regulation in the Prognosis of Chemotherapy Resistance and Aggressiveness of Breast and Lung Cancer. Adv. Clin. Med. Res..

[28] Fu Y.D., Huang M.J., Guo J.W., You Y.Z., Liu H.M., Huang L.H., Yu B. (2020). Targeting Histone Demethylase KDM5B for Cancer Treatment. Eur. J. Med. Chem..

[29] Zheng L., Wu Y., Shen L., Liang X., Yang Z., Li S., Li T., Shang W., Shao W., Wang Y. (2021). Mechanisms of JARID1B Up-Regulation and Its Role in Helicobacter Pylori-Induced Gastric Carcinogenesis. Front. Oncol..

[30] Wang Z., Tang F., Qi G., Yuan S., Zhang G., Tang B., He S. (2015). KDM5B Is Overexpressed in Gastric Cancer and Is Required for Gastric Cancer Cell Proliferation and Metastasis. Am. J. Cancer Res..

[31] Li Y., Chen L., Feng L., Zhu M., Shen Q., Fang Y., Liu X., Zhang X. (2019). NEK2 Promotes Proliferation, Migration and Tumor Growth of Gastric Cancer Cells via Regulating KDM5B/H3K4me3. Am. J. Cancer Res..

[32] Robertson A.G., Meghani K., Cooley L.F., McLaughlin K.A., Fall L.A., Yu Y., Castro M.A.A., Groeneveld C.S., de Reyniès A., Nazarov V.I. (2023). Expression-Based Subtypes Define Pathologic Response to Neoadjuvant Immune-Checkpoint Inhibitors in Muscle-Invasive Bladder Cancer. Nat. Commun..

[33] Wang D., Han S., Peng R., Jiao C., Wang X., Yang X., Yang R., Li X. (2016). Depletion of Histone Demethylase KDM5B Inhibits Cell Proliferation of Hepatocellular Carcinoma by Regulation of Cell Cycle Checkpoint Proteins P15 and P27. J. Exp. Clin. Cancer Res..

[34] Guo J.C., Liu Z., Yang Y.J., Guo M., Zhang J.Q., Zheng J.F. (2021). KDM5B Promotes Self-Renewal of Hepatocellular Carcinoma Cells through the MicroRNA-448–Mediated YTHDF3/ITGA6 Axis. J. Cell. Mol. Med..

[35] Schonfeld M., Averilla J., Gunewardena S., Weinman S.A., Tikhanovich I. (2022). Alcohol-Associated Fibrosis in Females Is Mediated by Female-Specific Activation of Lysine Demethylases KDM5B and KDM5C. Hepatol. Commun..

[36] Yan G., Li S., Yue M., Li C., Kang Z. (2021). Lysine Demethylase 5B Suppresses CC Chemokine Ligand 14 to Promote Progression of Colorectal Cancer through the Wnt/β-Catenin Pathway. Life Sci..

[37] Huang D., Xiao F., Hao H., Hua F., Luo Z., Huang Z., Li Q., Chen S., Cheng X., Zhang X. (2020). JARID1B Promotes Colorectal Cancer Proliferation and Wnt/β-Catenin Signaling via Decreasing CDX2 Level. Cell Commun. Signal..

[38] Metzler V.M., de Brot S., Haigh D.B., Woodcock C.L., Lothion-Roy J., Harris A.E., Nilsson E.M., Ntekim A., Persson J.L., Robinson B.D. (2023). The KDM5B and KDM1A Lysine Demethylases Cooperate in Regulating Androgen Receptor Expression and Signalling in Prostate Cancer. Front. Cell Dev. Biol..

[39] Turanli B., Grøtli M., Boren J., Nielsen J., Uhlen M., Arga K.Y., Mardinoglu A. (2018). Drug Repositioning for Effective Prostate Cancer Treatment. Front. Physiol..

[40] Agrawal P. (2015). Advantages and Challenges in Drug Re-Profiling. J. Pharmacovigil..

[41] Pantziarka P., Verbaanderd C., Sukhatme V., Capistrano R., Crispino S., Gyawali B., Rooman I., Van Nuffel A.M., Meheus L., Sukhatme V.P. (2018). ReDO_DB: The Repurposing Drugs in Oncology Database. Ecancermedicalscience.

[42] Zerbib M., Zelefsky M.J., Higano C.S., Carroll P.R. (2008). Conventional Treatments of Localized Prostate Cancer. Urology.

[43] Knipper S., Ott S., Schlemmer H.P., Grimm M.O., Graefen M., Wiegel T. (2021). Options for Curative Treatment of Localized Prostate Cancer. Dtsch. Arztebl. Int..

[44] Sayyid R.K., Klotz L., Benton J.Z., Ma M., Woodruff P., Satkunasivam R., Terris M.K., Wallis C.J.D., Klaassen Z. (2021). Active Surveillance in Favorable Intermediate-Risk Prostate Cancer Patients: Predictors of Deferred Intervention and Treatment Choice. Can. Urol. Assoc. J..

[45] Duarte D., Vale N. (2022). Antidepressant Drug Sertraline against Human Cancer Cells. Biomolecules.

[46] Lourenço T., Vale N. (2023). Pharmacological Efficacy of Repurposing Drugs in the Treatment of Prostate Cancer. Int. J. Mol. Sci..

[47] Sun W., Sanderson P., Zheng W. (2016). Drug Combination Therapy Increases Successful Drug Repositioning. Drug Discov. Today.

[48] FDA Antiviral Drug Advisory Committee Recommends Unanymously to Approve Entecavir for Hepatitis B. https://www.natap.org/2005/HBV/031505_02.htm.

